# Roles of Supplementary Motor Areas in Auditory Processing and Auditory Imagery

**DOI:** 10.1016/j.tins.2016.06.003

**Published:** 2016-08

**Authors:** César F. Lima, Saloni Krishnan, Sophie K. Scott

**Affiliations:** 1 Institute of Cognitive Neuroscience, University College London, London, UK; 2 Department of Experimental Psychology, University of Oxford, Oxford, UK

**Keywords:** supplementary motor area, auditory processing, auditory imagery, speech, music, sensorimotor mechanisms

## Abstract

Although the supplementary and pre-supplementary motor areas have been intensely investigated in relation to their motor functions, they are also consistently reported in studies of auditory processing and auditory imagery. This involvement is commonly overlooked, in contrast to lateral premotor and inferior prefrontal areas. We argue here for the engagement of supplementary motor areas across a variety of sound categories, including speech, vocalizations, and music, and we discuss how our understanding of auditory processes in these regions relate to findings and hypotheses from the motor literature. We suggest that supplementary and pre-supplementary motor areas play a role in facilitating spontaneous motor responses to sound, and in supporting a flexible engagement of sensorimotor processes to enable imagery and to guide auditory perception.

## Trends

Hearing and imagining sounds–including speech, vocalizations, and music–can recruit SMA and pre-SMA, which are normally discussed in relation to their motor functions.

Emerging research indicates that individual differences in the structure and function of SMA and pre-SMA can predict performance in auditory perception and auditory imagery tasks.

Responses during auditory processing primarily peak in pre-SMA and in the boundary area between pre-SMA and SMA. This boundary area is crucially involved in the control of speech and vocal production, suggesting that sounds engage this region in an effector-specific manner.

Activating sound-related motor representations in SMA and pre-SMA might facilitate behavioral responses to sounds. This might also support a flexible generation of sensory predictions based on previous experience to enable imagery and guide perception.

## From Action to Sound

**Figure 1:**
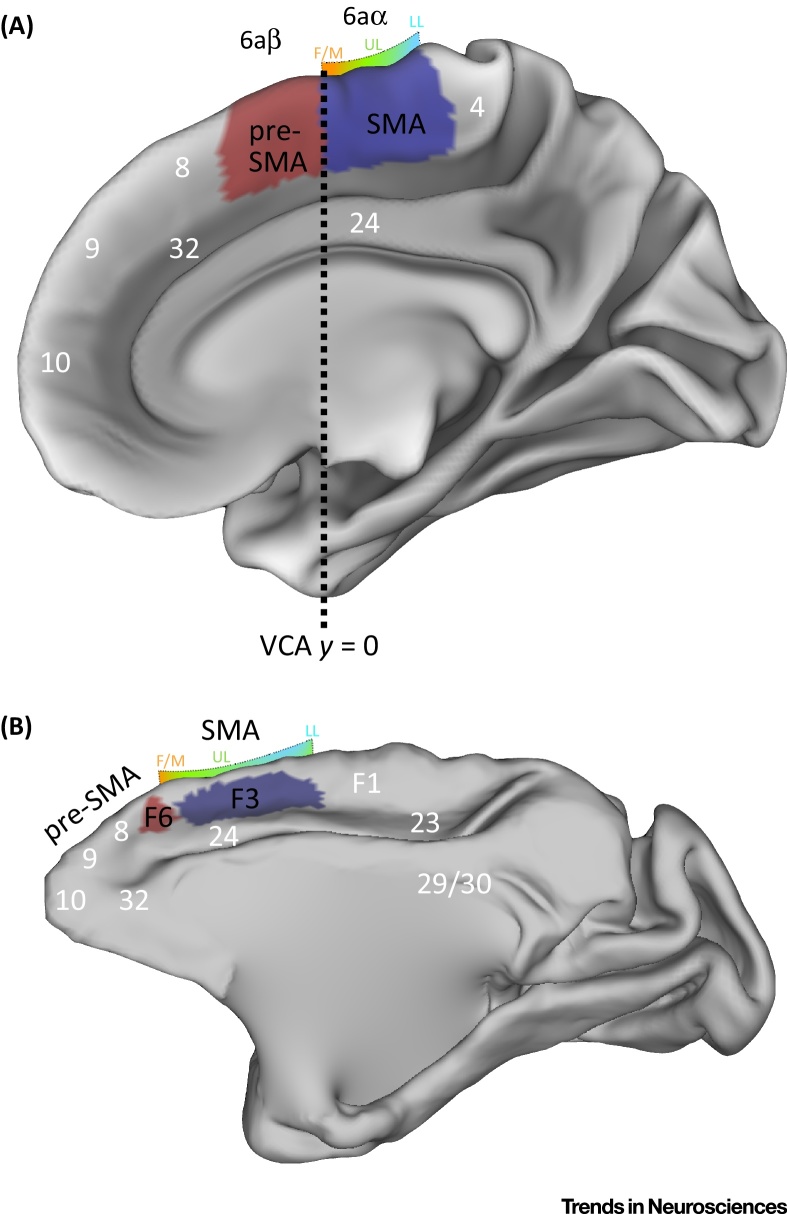
Anatomy of the Supplementary Motor Areas. (A) The medial surface of the right hemisphere of the human brain showing the locations of SMA (blue) and pre-SMA (red). SMA occupies the medial area 6aα, and pre-SMA is located anteriorly in medial area 6aβ. The broken line represents the vertical plane through the anterior commissure (VCA line, *y* = 0), typically considered as the border between pre-SMA and SMA. The maps were taken from the Human Motor Area Template (HMAT) [26], which uses both anatomical information and probability distributions estimating the likelihood of functional activation to characterize the shape, extent, and area of motor and premotor cortices. Area 4 corresponds to the primary motor cortex. (B) The medial surface of the macaque monkey brain showing area F3 (blue), which corresponds to SMA, and area F6 (red), which corresponds to pre-SMA. Area F1 corresponds to primary motor cortex. The human figure was generated from the Conte69 human surface-based atlas and the macaque figure was generated from subject F99 using Connectome Workbench software version 1.1.1. Anatomical parcellations of the macaque brain were sourced from the Markov *et al.*[118] atlas. Numbers in white represent locations of Brodmann's areas located in close proximity to SMA and pre-SMA. The gradients above SMA in the human and macaque brains illustrate the somatotopy in this region, with face/mouth (F/M), upper limb (UL), and lower limb (LL) representations depicted along an anterior-to-posterior orientation.

The premotor areas of the medial frontal cortex form a central node of the action network. More than a century ago, Horsley and Schafer [1] showed that electrical stimulation of these areas in monkeys produces movements of the trunk, proximal upper extremity, and head. The term ‘supplementary motor area’ was introduced in the 1950s to refer to this portion of the cortex [2], and although it was historically defined as a single area, more recent anatomical accounts suggest that it comprises two distinct fields: one anterior, the pre-supplementary motor area (pre-SMA), and one posterior, the supplementary motor area proper (SMA), with striking similarities in humans and monkeys 3, 4, 5 (Figure 1). These areas are known to be crucial for multiple aspects of motor behavior, including action preparation, initiation and selection of actions, motor learning, inhibition, conditional action, action control, and monitoring of action outcomes (6, 7, 8, for reviews 9, 10, 11].

**Figure 2:**
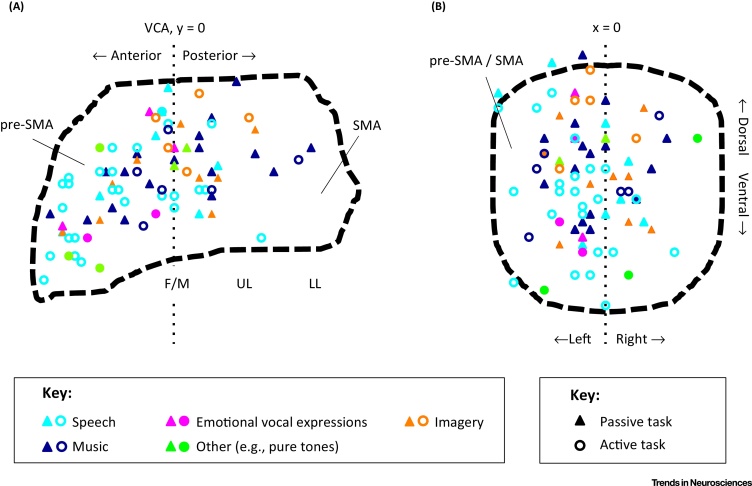
Key Figure: Responses in SMA and Pre-SMA During Auditory Processing and Auditory Imagery

In this review we address the candidate roles that SMA and pre-SMA play in auditory processing. These regions are commonly activated in auditory perceptual and auditory imagery studies, across a wide range of sounds including speech, nonverbal vocalizations, and music 12, 13, 14, 15, 16, 17. Notably, these responses are found even when the tasks do not involve overt motor components 14, 15, 16, 17 (Figure 2). The role of this activity remains an unresolved issue, possibly because SMA and pre-SMA are traditionally conceptualized as being linked to action-related processes, unrelated to audition. Likewise, in auditory cognitive neuroscience, these regions do not form part of typical auditory processing networks, in contrast to temporal, inferior parietal, lateral premotor, and prefrontal areas (Box 1).

Box 1 **Streams of Auditory Processing in the Human Brain**

As in the visual system, it is now well established that there are different anatomical and functional streams of processing in the auditory system. According to the dual-stream framework, sounds are processed in parallel along two largely segregated streams, an anteroventral and a posterodorsal one, both projecting from the primary auditory cortex (Figure I). These streams of processing engage with auditory information in distinctly different ways, and they play complementary roles in perception. The anteroventral pathway is important for the recognition and identification of auditory objects (‘what’), and it forms a hierarchically organized stream, involving parts of the auditory association cortex in the superior temporal gyrus and the inferior frontal gyrus. The posterodorsal pathway is important for aspects of sensorimotor integration and spatial processing (‘how/where’), involving posterior superior temporal fields, the inferior parietal cortex, motor and sensory areas, and the inferior frontal gyrus (for reviews 119, 120, 121).

**Figure I:**
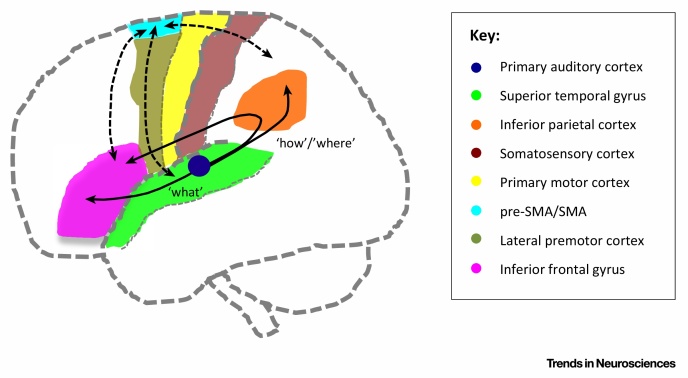
Streams of Auditory Processing in the Human Brain. Lateral view of the anteroventral and posterodorsal auditory processing pathways (unbroken arrows), and anatomical connections between SMA and pre-SMA and inferior frontal, temporal and parietal areas (broken arrows). The main anatomical systems involved in sound processing are shown in color. Projecting from the primary auditory cortex into anterior temporal and inferior frontal areas, the anteroventral stream decodes meaning in sounds (‘what’). The posterodorsal pathway supports sensorimotor and spatial processes (‘how’ and ‘where’), and it projects posteriorly into inferior parietal, sensory, motor, and inferior frontal areas.

The majority of studies on the perception of linguistic information in sound (e.g., intelligibility in speech as compared to non-intelligible sounds) show recruitment of anterior auditory ‘what’ fields, consistent with a move towards more abstract semantic representations in the temporal poles 122, 123. By contrast, posterior auditory ‘how’ areas seem to code both auditory and sensorimotor information. They are recruited not only during listening to sounds but also during speech production and silent articulation 124, 125, and in situations where articulatory behavior is modified via delayed auditory feedback [99]. In passive perceptual tasks, when heard sounds are meaningless (e.g., non-words, non-speech mouth sounds), posterior fields show greater activation than anterior ones [126]. There is also evidence that the posterodorsal pathway is strongly involved in the automatic, accurate control of auditory-motor actions. For example, listeners are able to automatically track subtle timing changes in sound sequences when they are asked to tap along to the sequences, and therefore engage posterodorsal systems; if they are asked about these changes, however, they fail to report hearing them, suggesting that the sensorimotor adjustments were controlled below the level of awareness [127].

Auditory processing is therefore a multifaceted phenomenon, involving multiple mechanisms that can interact in a complex manner. Understanding the role of SMA and pre-SMA in relation to these mechanisms might be especially relevant because action and sound are intimately related, and these regions are known to be connected to central nodes of the auditory network, namely inferior frontal, temporal, and parietal regions (Figure I) 20, 40, 44, 45, 46, 47, 48, 49.

## Anatomy and Motor Functions

SMA and pre-SMA are located anterior to the leg representation of the primary motor cortex, and they form part of the frontal agranular cortex, characterized by the lack of a granular layer IV 4, 11 (Figure 1). Falling within the superior frontal gyri, they roughly correspond to medial Brodmann area 6. Based on cytoarchitectonic and neurochemical differences, medial area 6 was later shown to comprise two distinct areas: SMA proper is located in medial area 6aα of Vogt and Vogt [18], and pre-SMA is located anteriorly in medial area 6aβ. Compared to SMA, pre-SMA shows a more-pronounced lamination and a clear demarcation of layer III from V [19], the cell density in the superficial layers is more homogeneous, and layer V is prominent and homogeneous [20]. In SMA, by contrast, layer III fuses with a dense layer Va [20]. The neurochemical footprint of these regions closely approximates these cytoarchitectonic differences, with clear stepwise changes in receptor binding densities at the boundaries between regions, and little variation within them [21]. Cytoarchitectonic and neurochemical similarities suggest that the SMA and pre-SMA in humans are homologous to macaque areas F3 and F6, respectively 10, 19.

The border between SMA and pre-SMA in humans is considered to be the vertical plane through the anterior commissure (VCA line, *y* = 0) 11, 22, and their inferior limit is at the level of the cingulate sulcus 23, 24. The anterior boundary of pre-SMA corresponds to the anterior end of the medial agranular cortex, bordering dysgranular cortex 4, 25. In neuroimaging work, because there are no macroanatomical landmarks for the anterior boundary, pre-SMA has been considered to extend to a virtual line passing through the genu of the corpus callosum [11]. An activation likelihood estimation (ALE) meta-analysis indicates that the functional limit is located 12 mm anterior to the VCA line at *z* = 70, and 26 mm anterior to the VCA line at *z* = 40 [26]. SMA and pre-SMA are anatomically and functionally distinct from other motor and premotor areas, namely from the dorsal and ventral premotor cortices in lateral Brodmann area 6 10, 27, 28, 29, 30.

SMA and pre-SMA differ in terms of their motor functions. SMA is tightly coupled with movement generation and control. Stimulation, neuroimaging, and lesion work in humans and non-human primates reveals a somatotopically organized map in this region along an anterior-to-posterior, face-to-legs gradient. Orofacial movements, including speech production, are associated with sites located anteriorly, at the border with pre-SMA, whereas upper and lower limb movements are associated with more posterior sites 1, 31, 32, 33, 34, 35, 36 (Figure 1). Similar somatotopic maps are found in motor and lateral premotor cortices, running in a ventral-to-dorsal, face-to-legs gradient 28, 30, 36, 37. In contrast to SMA, motor responses in pre-SMA can only be evoked using high current intensities, and the association between stimulation and movements is more variable. In humans and non-human primates, such evoked movements are typically slow, complex, and mainly involve orofacial and upper limb effectors 5, 11, 32, 34. Pre-SMA has been suggested to support higher-order aspects of action, including action preparation, the organization of movement sequences, the perception of objects that are potential targets of motor acts, and responses to sensory stimulation during cued movements 3, 5, 10, 38. The distinct functional properties of SMA and pre-SMA are potentially related to their patterns of connectivity.

Connectivity analyses show that SMA, but not pre-SMA, can directly influence motor output via its direct connections to the primary motor cortex and spinal cord 20, 22, 39, 40. This region is also connected with the posterior premotor and cingulate areas, and parietal regions [20]. By contrast, pre-SMA is richly connected to prefrontal, anterior premotor, and cingulate areas, as well as to the medial parietal cortex 20, 22, 41. Given its position between the prefrontal and motor areas, pre-SMA can be conceptualized as an area that integrates information about action plans, motivation, and objects, which can be used for action initiation and control [10]. Both SMA and pre-SMA receive basal ganglia projections 40, 42, 43, with pre-SMA connecting to more anterior sites of the striatum than SMA, as indicated by *in vivo* imaging tractography in humans 22, 43. Such corticostriatal connections have been suggested to be part of a wider network that, through the thalamus, projects back to the cortex, and supports different aspects of motor control [40]. Furthermore, evidence from tracer injection in non-human primates also indicates that pre-SMA is connected to SMA [20].

Importantly, there are structural and functional connections between SMA, pre-SMA, and regions that are considered to be central for sound and speech processing (see Figure I in Box 1). Post-mortem dissection and imaging tractography evidence from humans has revealed a direct pathway, the frontal aslant tract, directly connecting SMA and pre-SMA with the opercular part of inferior frontal gyrus 40, 44, which corresponds to Broca's area in the dominant hemisphere and is a core component of the posterodorsal auditory pathway. The frontal aslant tract is observed in both hemispheres, but diffusion tractography indicates that it is left-lateralized in most right-handed individuals [44]. A study of primary progressive aphasia found that reductions in the structural integrity of this tract relate to performance in a verbal fluency task [45], suggesting an involvement in language production. Pre-SMA also has connections to the temporal lobe. Tracer injection studies in non-human primates have revealed connections to the anterior superior temporal sulcus 20, 46, and functional connectivity analyses in humans reveal connections to the superior temporal gyrus 47, 48. Both SMA and pre-SMA are connected with the parietal lobe, including inferior parietal sites 20, 48, 49, which are part of the posterodorsal pathway (see Figure I in Box 1).

## Auditory Information in SMA and pre-SMA

Consistent with their patterns of connectivity, SMA and pre-SMA are recruited during auditory processing (Figure 2, Key Figure). For speech perception, functional neuroimaging studies have reported activity in response to syllables 50, 51, words 52, 53, and sentences 54, 55, 56, 57, 58, 59. Such responses seem to be modulated by the difficulty of speech processing and comprehension. In pre-SMA, activity is inversely correlated with speech intelligibility as manipulated by background noise; in other words, responses are stronger when the speech signal is less intelligible and comprehension is more challenging 15, 60. In an ALE meta-analysis of functional magnetic resonance imaging (fMRI) and positron emission tomography (PET) studies, Adank [61] observed that, in addition to anterior insula fields, pre-SMA was the only area of the motor and premotor cortices recruited during the processing of distorted/less-intelligible speech as a result of background noise, regional accents, or speech rate differences.

The involvement of SMA and pre-SMA is not limited to the perception of speech: activity is observed during listening to familiar 13, 17 and unfamiliar music 62, 63, as well as during the perception of action sounds (e.g., drinking from a straw) [64], and sequences of tones 65, 66. Speech melody and nonverbal emotional vocalizations, such as laughter and crying, also evoke responses in SMA and pre-SMA 12, 14, 16, 67, which suggests that auditory responses in these regions extend to the processing of socio-emotionally salient sounds (Box 2).

Box 2 **Emotional Voices in SMA and Pre-SMA**

The processing of nonverbal vocal emotional cues is key for vocal communication. These cues include purely nonverbal vocalizations, such as laughter or crying, and modulations of the tone of voice while speaking (emotional prosody). Several studies indicate that listening to vocal emotional cues, and inferring their meaning, recruit SMA and pre-SMA. In a study of nonverbal vocalizations, including laughter, triumph sounds, screams, and disgust sounds, Warren *et al.* [14] showed that responses in SMA and pre-SMA are significantly modulated by the type of sound, in a passive listening task not involving overt motor responses. These modulations were further related to broader affective dimensions of arousal, with high-arousal vocalizations activating these regions more strongly than low-arousal ones. While these findings were taken to reflect a spontaneous impulse to respond to the emotional expressions of others, more recent work showed that responses in these regions might also contribute to perceptual processes. McGettigan *et al.* [16] reported activations in pre-SMA during passive listening to voluntary social-type laughter and spontaneous laughter, and found that the magnitude of these responses predicted performance in a post-scanner behavioral authenticity detection task. Consistent with a role in the perception of auditory expressions, Bestelmeyer *et al.* [12] showed that pre-SMA responses are associated with greater discrimination between expressions taken from continua of anger and fear nonverbal vocalizations, even after regressing out the low-level acoustic features of the expressions.

Activations in SMA and pre-SMA can also be obtained for the perception of emotional prosody signals during passive listening [140] and during explicit evaluations of emotional authenticity [141]. There is suggestive evidence that this might be modulated by training. Kreifelts *et al.* [142] showed that 4 weeks of non-verbal emotion communication training produces selective changes in a network of regions including bilateral pre-SMA. This was observed in a task requiring valence judgments of multimodal stimuli comprising speech prosody and facial emotional cues.

The links between SMA and pre-SMA and vocal emotional processing possibly reflect a broader association between these regions and socio-emotional variables. Resections of the pre-SMA correlate with impairments in the ability to infer intentions (mentalizing) [143], and a transcranial magnetic stimulation study showed that stimulation of SMA modulates the perceived valence of emotional visual stimuli [144]. The interactions between motor-related activity in SMA and pre-SMA and socio-emotional factors could be supported by direct and indirect anatomical connections between these regions and emotional systems including cingulate regions and the limbic system 40, 144.

SMA and pre-SMA are consistently implicated in auditory imagery as well, when participants are instructed to generate auditory mental images in the absence of sensory input (Box 3). This includes imagery of speech, such as syllables and words 68, 69, and imagery of music (13, 70; for a meta-analysis [71]). Indeed, these regions arguably predominate over sensory cortex in such auditory imagery tasks: studies directly contrasting music listening and music imagery showed that SMA and pre-SMA, unlike motor and lateral premotor cortices, are recruited more strongly for imagery than for listening, while the superior temporal gyrus is recruited more strongly for listening than for imagery 13, 72, 73.

Box 3 **Auditory Imagery**

Auditory imagery refers to the process by which individuals generate auditory information in the absence of sound perception, such as when we imagine the voice of a friend or the sound of a familiar song. Auditory imagery can be reported to be so vivid that it resembles the experience of hearing, and to be as accurate as auditory representations arising from sensory input [115]. Auditory mental images cannot be directly observed or measured, but their properties have been inferred using strategies such as self-report 13, 80, 114, and performance-based tasks in which participants provide judgments that require engaging in imagery (e.g., judging the pitch of words taken from familiar tunes in the absence of auditory input [73]; or judging whether a final note of a scale is mistuned when the initial notes were played but the remaining ones had to be imagined [115]). An approach often used in functional neuroimaging and electrophysiological research consists of comparing conditions where participants generate auditory imagery with conditions where they are presented with auditory stimuli, or engage in tasks not involving imagery 13, 69, 72, 73, 81, 115.

Studies focusing on voluntary imagery of music and speech reveal the recruitment of a network of brain regions including the superior temporal gyri, parietal, motor, and lateral premotor cortices, the inferior frontal gyrus, and SMA and pre-SMA (13, 68, 69, 70, 72, 73, 80, 81; for a meta-analysis [71]). This network substantially overlaps with the network recruited during auditory perception, including auditory areas. Auditory cortex activity during imagery is thought to reflect the instantiation of sound-like representations as controlled by higher-order cortical mechanisms 13, 73, contributing to the phenomenological experience of ‘hearing’.

Little is known about involuntary forms of auditory imagery, such as ‘earworms’ (the experience of having music looping into one's head). Farrugia *et al.* [145] reported associations between self-report frequency of earworms and cortical thickness in frontal and temporal cortices, and in the anterior cingulate and angular gyrus. The length of earworm episodes correlated with cortical thickness in anterior pre-SMA, and affective evaluations of this form of imagery correlated with grey matter volume in temporal-polar and parahippocampal cortices.

Studies on mental imagery are typically confined to a single modality (e.g., auditory, visual, motor), but there is some evidence suggesting that, while superior temporal areas play an auditory-specific role, other components of the auditory imagery network, namely motor and premotor systems, might be also engaged by other forms of imagery 71, 80. More research directly contrasting different forms of imagery will be needed to address this question.

Not all forms of auditory imagery are voluntary: people often experience involuntary musical imagery, a phenomenon colloquially termed ‘earworms’ (Box 3); and auditory verbal hallucinations form another example of auditory images that are experienced as uncontrollable and as arising from an external source. Pre-SMA might be important for the sense of volitional control (intentionality) over auditory images. In an fMRI study comparing auditory verbal hallucinations and voluntary imagery of the same voices, pre-SMA activity was stronger during imagery than during hallucinations, both when hallucinations were compared with imagery within the same group of patients and when they were compared with imagery in healthy controls [74]. Work on auditory hallucinations in healthy controls (‘nonclinical voice hearers’) has shown that pre-SMA is recruited both during voluntary imagery and hallucinations; notably, however, while during imagery pre-SMA becomes activated before inferior frontal and temporal fields, during hallucinations it becomes activated at the same time as these areas [75], suggesting an impaired control of auditory and motor processes by pre-SMA in hallucinations.

Although SMA and pre-SMA activity is obtained during auditory perceptual and imagery tasks, it could simply reflect task-related motor or general cognitive processes, such as button presses or response selection, or it could reflect an epiphenomenal response, unrelated to audition. However, there is strong evidence for such activity during passive listening to different types of sounds, when no movements or response selection are involved 14, 15, 16, 17, 54, 76, 77 (Figure 2). Indeed, even when movements are involved, such as during overt task performance, activations in these regions are can be seen over and above the neural responses associated solely with motor performance 12, 67, 78. SMA and pre-SMA activity further varies with auditory-motor experience; studies on expert musicians indicate that auditory-motor training modulates responses to sound in these regions, pointing to a specific association beyond task effects [79] (Box 4). Consistent with a direct role for SMA and pre-SMA in auditory processes, inter-individual differences in the structure and function of these regions can predict outcomes in auditory imagery and auditory perception tasks. In a voxel-based morphometry study, larger grey matter volume in SMA was shown to predict higher perceived vividness of mental images during auditory imagery [80]. In a related vein, evidence from fMRI indicates that larger functional responses in SMA during imagery of familiar melodies correlates with higher perceived vividness of auditory images [81]. In auditory perception, the magnitude of functional responses in pre-SMA during passive listening to posed and spontaneous laughs was shown to correlate with better ability to categorize laughter authenticity in an off-line behavioral task [16]. In a task requiring participants to judge the direction of temporal changes in frequency-modulated sounds (‘speeding-up’ or ‘slowing-down’), while ignoring simultaneous pitch changes in the same sounds, pre-SMA responses were linked with perceptual sensitivity to discriminate temporal changes, as well as with reduced susceptibility to distortions in temporal judgments induced by pitch changes [82]–in other words with a better ability to focus on the task-relevant features of the stimuli (temporal changes) and ignore the irrelevant ones (pitch changes). These findings provide initial support for the hypothesis that SMA and pre-SMA directly influence a variety of auditory processes. However, determining the nature of this contribution, and whether it represents a modulatory or a central contribution, requires further investigation. Studies of patients with SMA and pre-SMA lesions, and using techniques such as transcranial magnetic stimulation (TMS), will be important for this. TMS studies have shown that disrupting activity in primary motor and lateral premotor fields impairs the discrimination of speech sounds [83] and vocalizations [84]. This approach could be extended to examine the roles of SMA and pre-SMA in the perception and imagery of different types of auditory information (see Outstanding Questions).

Box 4 **Individual Differences and Plasticity in SMA and Pre-SMA**

The structure and function of SMA and pre-SMA can vary between individuals as a consequence of motor learning and expertise. Structural changes can occur over relatively short timescales–for instance, grey-matter increases in these regions were observed over 6 weeks of practicing a complex motor skill (dynamic balance task), and these structural changes correlated with changes in functional connectivity over time 128, 129. Structural changes in SMA have also been found in specialized groups of participants who completed intense long-term sensorimotor training, such as ballet dancers [130] and athletes 131, 132. Directly relevant to auditory processing, a large study of musicians [133], who spend years developing a repertoire of specific motor actions in relation to their sensory consequences, revealed a positive correlation between musical expertise and increased grey matter volume in SMA.

In addition to structural changes, motor skill learning can modulate functional responses in SMA and pre-SMA–in the context of initial stages of learning, activity has been shown to decrease in pre-SMA and to increase in SMA as performance improves ([134] for review). Long-term auditory-motor experience also correlates with functional changes in SMA. For instance, opera singers show greater activity in left SMA during overt singing than novices do [135]. These changes might explain why musicians perform better in new motor tasks. While they might be able to automatically recruit SMA and pre-SMA for motor performance and sensorimotor integration, independently of the complexity of the task, the engagement of non-musicians might be more strongly constrained by task difficulty 136, 137.

Not only does functional activity in SMA and pre-SMA appear relevant to action execution but it may also shape the auditory perception of learnt actions. When listening to music, musicians recruit pre-SMA more strongly than non-experts, in a way that does not seem to reflect a general unspecific effect–it is selective for music that they can produce [79]. Furthermore, higher sensitivity to beat perception in music relates to a stronger engagement of SMA [138]. Interestingly, SMA activity during auditory processing can be modulated by other forms of sensorimotor expertise in addition to musical expertise. Athletes who play basketball or tennis show stronger activity in SMA during listening to sport sounds, such as a racquet hitting a ball, as compared to non-sport sounds, such as paper crumpling. In addition, even within the category of sport sounds, SMA activations are stronger for sounds related to the sport that the athletes can play than to sounds related to a sport that they cannot play [139].

In terms of lateralization of activity, many studies report bilateral involvement of SMA and pre-SMA during auditory processing 52, 64, 80, 85, and peak activations can be found in both hemispheres. However, left hemisphere responses predominate, if we take the relative number of peak activations reported in the left and the right hemisphere as an indication of lateralization (Figure 2). This may mean that there is more to learn about hemispheric asymmetries within these regions (see Outstanding Questions). Given that assigning midline responses to the left or right hemisphere might be difficult using fMRI, future research will benefit from also using other approaches to address this question, such as single-cell recordings or studies of patients with unilateral lesions.

Figure 2 shows that peak auditory and imagery responses in SMA and pre-SMA are not evenly distributed across the surface of these regions. They are primarily found in pre-SMA, and in a cluster around the border between pre-SMA and SMA; responses are more rarely found in posterior SMA. Interestingly, the area around the border between the two regions is consistently associated with the production of orofacial movements, vocalizations, and speech 31, 32, 33, 35. Lesions in this area can selectively produce aphasia, without motor limb impairments [31], and affect the production of vocalizations in squirrel monkeys [86]. Thus, in addition to engaging pre-SMA, auditory information engages SMA fields that are also involved in the generation and control of movements related to vocal production. This is suggestive of effector-specific motor recruitment during auditory processing and auditory imagery. Evidence for the spatial overlap between perception and production in these regions comes from several studies directly comparing the two conditions 14, 16, 56, 64 (Box 5). It is also interesting to note that the frontal aslant tract, that connects the inferior frontal gyrus with medial frontal areas and has been suggested to play a role in language production [45], terminates at the border area between SMA and pre-SMA [40]. The possible involvement of this tract in auditory perception and imagery has yet to be determined.

Box 5 **Linking Auditory Perception and Production in SMA and Pre-SMA**

There is strong evidence that SMA and pre-SMA have sensorimotor properties, and respond to both the perception and execution of actions. Neuroimaging studies report overlapping activity in the right and left pre-SMA during action perception and action production, as revealed by an activation likelihood estimation (ALE) meta-analysis [146]. Importantly, this overlapping activity does not simply reflect the averaging over voxels that respond to perception and production at a group level–instead, shared responses were shown to consistently emerge at a single-subject level [147]. Further evidence comes from single-cell recordings. Mukamel *et al.* [148] recorded extracellular activity in human medial frontal and temporal cortices, and found that a significant number of neurons in SMA responded during the execution and observation of hand grasping actions and facial emotional expressions.

Many studies focus on perception–production couplings in the visual domain, but there is evidence for similar couplings in the auditory domain. Gazzola *et al.* [64] observed overlapping SMA activity during the execution of motor actions and during passive listening to sounds associated with those actions. Overlapping activity has also been found in SMA and pre-SMA during listening to emotional vocalizations such as laughter, and during the production of related orofacial movements 14, 16. Studies on expert musicians suggest that these sensorimotor associations might be modulated by learning and experience–while both listening to and producing music lead to overlapping activity in SMA, this region is more strongly engaged by musicians than by non-musicians [149].

An emerging literature suggests that sensorimotor links in SMA and pre-SMA extend to speech processing. In an ALE meta-analysis of studies examining the neural bases of speech production and speech perception in difficult listening conditions, Adank [61] reported that SMA and pre-SMA are part of both speech comprehension and production networks. These regions are additionally involved in monitoring auditory feedback during speech production, particularly in demanding or noisy speaking conditions 100, 150. Gauvin *et al.* [56] observed that monitoring speech errors in both speech perception and production tasks leads to activity in the left pre-SMA. This suggests that SMA and pre-SMA are sensorimotor resources that can be recruited when speech processing becomes challenging, perhaps because they can be used to generate and constrain predictions about the sensory input.

The overlap of perceptual and action mechanisms in SMA and pre-SMA suggests that these regions are part of the ‘mirror neuron’ system. Research on mirror neurons has traditionally focused on ventral premotor and inferior parietal areas, but some studies have begun to discuss SMA and pre-SMA in the context of this system 64, 146, 147, 148. Important claims of the mirror neuron theory remain controversial, however, including the ideas that these neurons code action goals and directly support the understanding of actions and intentions [151].

## Candidate Functions of SMA and Pre-SMA Responses

There is no consensus position on the roles of SMA and pre-SMA responses in auditory processing and imagery. When such responses are discussed, they have been linked to a variety of processes. Timing functions have been suggested for perceptual tasks requiring evaluations of temporal aspects of auditory stimuli [82], or for stimuli varying in the sequential predictability and rhythmic regularity that they afford 66, 76, 87, 88, 89. SMA and pre-SMA, together with the cerebellum and the basal ganglia, have in fact been considered to form the substrates for a ‘temporal processing’ network (for reviews 90, 91, 92, 93). By contrast, sub-vocalization and articulatory/motor roles for SMA and pre-SMA have also been suggested, in the context of studies on imagery and perception of speech and music, because these stimuli can be easily reproduced using the vocal apparatus 13, 15, 69, 73. When tasks emphasize detecting abnormalities in the signal, such as speech errors or noise-related interruptions in speech 56, 94, SMA and pre-SMA have been linked to monitoring functions. There are few attempts to link these different proposals, and a unified framework that accounts for perceptual and imagery processes across different sound categories has not been proposed.

Our starting point in developing a model of SMA and pre-SMA roles in auditory processing is our finding that peak auditory and imagery responses cluster in pre-SMA, and extend to the boundary between pre-SMA and SMA (Figure 2). This might indicate the involvement of higher-order control of action-related processes, together with coupling to vocal production, because more complex and controlled aspects of motor behavior are linked with pre-SMA, while a more direct role in action execution is linked with SMA 9, 10, 11. Several higher-order functions have been attributed to pre-SMA in humans and non-human primates, namely the integration of sensory and motor information (sensorimotor processing), planning of actions, focus on current goals while inhibiting interference from irrelevant cues, and intentional initiation of action 9, 10, 95, 96. In the context of speech production, for example, fMRI and PET studies have shown that complex processes such as word selection and propositional speech recruit anterior pre-SMA sites, whereas simple articulatory movements recruit more posterior sites 97, 98. In the context of temporal processing, tasks emphasizing perceptual aspects of the stimuli (e.g., making temporal judgments of auditory stimuli) tend to recruit pre-SMA, whereas tasks emphasizing motor responses (e.g., tapping along to a rhythm) recruit relatively more posterior sites [93].

We argue that pre-SMA and the boundary area between pre-SMA and SMA could provide a mechanism for linking auditory information with related motor programs, and that this sensorimotor engagement could be modulated by controlled processes as part of a wider network involving prefrontal, auditory, and other sensorimotor systems. We propose two parallel candidate roles for SMA and pre-SMA: motor facilitation, which might provide a mechanism for supporting spontaneous behavioral responses to sounds; and a more controlled and flexible process, in that motor programs would be retrieved to generate and exploit sensory expectations that would enable imagery and optimize perceptual processes. Aspects of auditory perception and auditory imagery may be characterized by such auditory–motor interactions.

### Auditory–Motor Interactions

Sound and action are inherently linked. Our auditory experience can be conceptualized as a sensorimotor interplay: the sounds that we hear reflect actions, and the sounds that we make result from actions. We learn to associate actions, such as speaking or singing, with their sensory correlates through experience. This establishes bidirectional links between actions and the corresponding sounds. One illustration of these associations is the role of auditory information in the control of speech production. Manipulations of the online perception of self-produced speech, for instance via delayed auditory feedback, detrimentally affect the act of speaking, and modulate activity in sensory and motor networks 99, 100. Music training with auditory rhythmic cueing improves motor functions in Parkinson's patients (gait kinematics), and such auditory cueing-related benefits extend beyond gait to motor timing tasks and to auditory time perception (duration discrimination of pure tones and detection of misaligned beats in music) [101]. SMA and pre-SMA are well placed, anatomically and functionally, to support interactions between motor and sensory processes in both an overt and a covert manner. Pre-SMA is recruited during auditorily cued actions, and such recruitment is stronger when participants learn to associate auditory cues with specific actions than when they listen to the same cues and produce the same actions without a specific association between them [102], suggesting a role in sensorimotor integration. SMA and pre-SMA are also important for motor imagery 103, 104, possibly relying on the mechanisms that also support overt motor execution [105]. Activating motor programs in these regions can thus serve action execution, as well as cognitive processes that do not involve overt motor behavior but might benefit from motor-related processing. Furthermore, motor information can modulate sensory processes via efference copies of the motor programs, sent in parallel to sensory systems and to relevant effectors, and SMA has been pointed out as a likely source of this modifying activity [47].

### Spontaneous Engagement and Motor Facilitation

We suggest that SMA and pre-SMA are part of the network that supports the spontaneous engagement of motor programs related to sounds. Some sounds, owing to their rhythmical patterns or their social and motivational salience, elicit motor responses, such as singing, tapping, dancing, or vocal alignment/contagion. Infants aged 5–24 months spontaneously coordinate body movement to music, with the degree of coordination correlating with positive affect [106]. Vocalizations such as laughter can be primed solely by listening to someone laugh, and are strongly primed by the presence of other people [107]. During spoken interactions, people spontaneously synchronize breathing, coordinate turn-taking, and imitate others’ speech patterns, for example in terms of intonation, speech rate, and accent 108, 109, 110. Such propensity to respond to rhythmic, social, and emotional auditory information might promote social convergence, learning, coordination, and affiliation.

Electrical stimulation of SMA and pre-SMA can elicit a subjective ‘urge’ to move [32], and neural oscillations and fMRI studies indicate that these areas are engaged during passive listening and motor synchronization to auditory rhythms 76, 111. Consistent with this, SMA and pre-SMA are also activated during passive listening to music, and responses are stronger for rhythmic sequences as compared to random sequences [87], and for familiar music as compared to random sequences [77]. In an fMRI study, Silva-Pereira *et al.* [17] showed that SMA is more strongly recruited by familiar than unfamiliar music, and by music that listeners like as compared to music that they dislike. This suggests that the readiness to respond with motor activity to music might be affected by how much we can predict upcoming events based on previous knowledge, and that motivational factors might play a modulatory role as well. SMA and pre-SMA are also engaged during listening to emotionally salient human vocalizations, including laughter [14] (Box 2), which are primed by both social and emotional factors.

### Controlled and Flexible Processing

In parallel with a role in facilitating spontaneous behavioral responses, SMA and pre-SMA are well placed for supporting a more controlled engagement of sensorimotor processing during auditory perception and imagery. Pre-SMA, in particular, as an interface area between prefrontal and motor systems, could support the initiation and control of sensorimotor engagement, integrating information about goals, context, and motivation in that process. This would provide a mechanism for generating internal estimates of the sensory correlates of sound-related actions, and these estimates would enable dynamically updated sensory expectations that could flexibly contribute to various aspects of perception and imagery. In the context of imagery, estimates of the sensory correlates of candidate actions would be the substrate of the subjective experience of ‘hearing’. This is consistent with the observation that responses in the superior temporal gyrus and in pre-SMA and SMA are both seen in auditory imagery studies 13, 69, 71, and with evidence linking individual differences in the structure and function of SMA with higher vividness of auditory images 80, 81. Further evidence for a role of pre-SMA in controlled/intentional aspects of imagery comes from studies showing that this region is more strongly engaged, and is activated before auditory regions, during voluntary imagery as compared to (involuntary) auditory verbal hallucinations 74, 75. The involvement of SMA and pre-SMA in activating relevant motor programs (motor simulation) during auditory imagery, and the potential contribution of this for estimating and predicting auditory representations, was recently suggested in the context of imagery of speech [69].

In auditory perception, the same controlled engagement of sensorimotor processes might contribute to guiding and optimizing different perceptual processes. Sensory expectations generated based on previous sensorimotor experience might orient attention to goal-relevant features of the auditory input, suppress irrelevant ones, reconstruct missing information, and monitor the flow of incoming events. These ideas resonate with the ‘emulation’ framework discussed in relation to action control, and generalized to motor imagery, perception, and prediction 112, 113. Converging mechanisms for auditory imagery and auditory perception in SMA and pre-SMA have been suggested 13, 73, 80. For example, individuals reporting highly vivid auditory imagery show more specific neural representations of sounds during auditory perception in the same SMA peak that is associated with imagery [80]. Behavioral and electrophysiological studies further show that generating internal representations and expectations of auditory events benefits vocal production [114] and auditory perceptual processes 115, 116. In an fMRI study examining the perception of artificially degraded words, Shahin *et al.* [94] found stronger pre-SMA responses when participants subjectively experienced physically interrupted words as continuous, as compared to when they had the same experience of continuity for actual physically continuous words. By providing a flexible mechanism for engaging sensorimotor processes, this region might thus be involved in ‘repairing’ degraded auditory information. Such repair mechanism could relate to findings on other forms of degraded/distorted speech: pre-SMA is more strongly activated during the processing of time-compressed as compared to normal speed speech [85], and for speech in higher as compared to lower levels of background noise [15].

Auditory perception involves multiple streams of processing, and auditory objects can often be categorized via obligatory hierarchical processing in the anteroventral ‘what’ auditory pathway (Box 1). By contrast, the engagement of sensorimotor mechanisms in SMA and pre-SMA likely does not reflect early perceptual processing for meaning. Instead, it could be part of a parallel, more flexible stream of processing, and the magnitude of its contribution to perception and imagery could be a function of previous sensorimotor experience relevant to the listening/imagery context, difficulty of sensory-based processing, and task goals. A stronger contribution could be expected when previous experience enables the generation of rich and fine-grained sensory expectations, when the representation of the sensory input is degraded/distorted or absent (imagery), and when the task benefits from optimal sensorimotor integration or requires explicit/attentive access to auditory representations. Different lines of work provide initial support to this idea. Studies on expertise indicate that auditory-motor experience modulates SMA and pre-SMA responses during auditory processing [79] (Box 4), possibly reflecting a facilitated generation of sensory expectations. Stronger responses are obtained during auditory imagery, when there is no sensory input, as compared to listening 13, 72, 73; and they are stronger during listening in difficult conditions, when sensory input is degraded or distorted, than when the sensory input is relatively intact 15, 85, 94. Finally, there is a positive association between the structure and activation of SMA and pre-SMA and performance on a variety of explicit auditory tasks, requiring not only accessing temporal representations of the stimuli, but also judging other aspects such as socio-emotional categories and vividness of mental images 12, 16, 80, 81, 82.

An emerging theme from our review of the literature is that auditory information often engages SMA fields that are also linked to vocal production. Our vocal apparatus is arguably our primal, original instrument for making sounds. Because of the multifaceted and flexible nature of vocal production, the representation of these effectors could form a representational space within which we could generate sensorimotor estimations of different properties of sound. This could include the identity of linguistic information, and inferences about nonverbal signals such as voices and music and how they unfold in time. It could also include aspects related to rhythm, timing, or sequencing of auditory events, because these are all central features of vocal production. For instance, Wolfensteller *et al.* [117] showed that SMA is recruited during rhythm predictions in visual sequences of abstract stimuli, as compared to predictions about positions and objects. This finding in the visual domain might reflect the correspondence between the emphasis of the task (rhythm) and a feature that is central for a given effector (articulatory system). Consistent with this, tracking temporal and rhythmic aspects of a variety of auditory and visual stimuli has been shown to recruit the boundary area between pre-SMA and SMA [93], similarly to vocal production.

## Concluding Remarks

Auditory stimuli, both heard and imagined, activate SMA and pre-SMA. The broad evidence for these activations suggests that we in auditory cognitive neuroscience need to conceptualize these regions as part of the functional neuroanatomy of auditory processing, and to account for the variety of listening and imagery contexts in which they are involved. This matters because SMA and pre-SMA are not typically included in models of auditory processing and, when the roles of the motor and premotor systems are considered, the focus is primarily on lateral fields. We argue that SMA and pre-SMA support the activation of sound-related motor representations during auditory perception and auditory imagery, and that this activation can be modulated by controlled processes. According to our hypothesis, these regions mediate spontaneous motor responses to sound, and support a more controlled generation of sensory predictions based on previous sensorimotor experience, predictions that can be flexibly exploited to enable imagery and optimize a variety of perceptual processes. Future research will need to delineate the precise relationship between SMA and pre-SMA and the regions traditionally considered to the part of auditory streams of processing (Box 1). Another crucial aspect of future developments will be to refine our understanding of which aspects of auditory processing, context, and current goals/task are more directly dependent on the functions of SMA and pre-SMA.

Outstanding Questions Are there hemispheric asymmetries in auditory processing in SMA and pre-SMA? Does this differ depending on the nature of the sound, and on whether it is heard or imagined?

Does the spatial overlap of activity across auditory perception, imagery, and sound production in SMA and pre-SMA reflect the engagement of the same neural populations and mechanisms? Do these regions contain both mechanisms specialized for action as compared to perception (and vice versa) and mechanisms shared across modalities?

Do responses in SMA and pre-SMA during auditory processing and imagery correlate with the ability to produce the corresponding sounds?

How do SMA and pre-SMA interact with the nodes of the anteroventral and posterodorsal streams of auditory processing in typical as well as in atypical conditions, such as auditory verbal hallucinations?

How does damage or stimulation of SMA and pre-SMA affect auditory processing and imagery? Are these possible effects modulated by task conditions, sound category, and by the level of degradation of the sensory input?
