# Supplementary material for: Roles of Supplementary Motor Areas in Auditory Processing and Auditory Imagery
Source: Trends Neurosci. 2016 Aug;39(8):527–42. doi: 10.1016/j.tins.2016.06.003 (PMC5441995; doi:10.1016/j.tins.2016.06.003)
Supplement: Supplementary file 1 [file mmc1.docx]

**Supplemental Information**

Roles of Supplementary Motor Areas in Auditory Processing and Auditory Imagery

César F. Lima^1^, Saloni Krishnan^2^, and Sophie K. Scott^1^

^1^Institute of Cognitive Neuroscience, University College London, London, UK

^2^Department of Experimental Psychology, University of Oxford, Oxford, UK

Correspondence: [sophie.scott@ucl.ac.uk](mailto:sophie.scott@ucl.ac.uk)

**Table S1**

Neuroimaging studies included in Figure 2. We selected studies using functional magnetic resonance imaging (fMRI), voxel-based morphometry (VBM) and positron emission tomography (PET). They were included if they reported peak activations in supplementary or pre-supplementary motor areas (SMA/pre-SMA) during auditory stimulation or during explicit instructions to imagine sounds. The selection was based on the authors’ knowledge of the literature, and on an extensive search in the PubMed database (www.pubmed.com), using strings such as ‘supplementary motor area AND (speech OR music OR auditory imagery OR voice OR auditory OR vocalizations OR sounds)’, and ‘supplementary motor area AND fMRI AND (speech OR music OR auditory imagery OR voice OR auditory OR vocalizations OR sounds)’. We only included studies that were published in English and that investigated samples of healthy adult participants. The included peaks had to: result from whole-brain level analyses; reflect a clear categorical comparison or parametric effects of auditory-relevant experimental measures; and result from within-subject contrasts, not from group comparisons. We considered only paradigms not involving motor responses in contrast-relevant trials (e.g., passive listening), or paradigms involving active tasks, as long as the peaks obtained with active tasks resulted from comparisons between conditions with comparable motor demands. When multiple SMA/pre-SMA clusters were obtained in the same study, they were all considered for inclusion in Figure 2 only if they resulted from orthogonal contrasts; if they resulted from a single contrast, we only selected the statistically strongest peak.

| # | Authors | Year | N | Method | Category | Task | MNI Coordinates | | |
| --- | --- | --- | --- | --- | --- | --- | --- | --- | --- |
|  |  |  |  |  |  |  | x | y | z |
| 1 | Adank & Devlin | 2010 | 22 | fMRI | Speech | Active | 0 | 12 | 60 |
| 2 | Adank et al. | 2013 | 32 | fMRI | Speech | Active | -2 | 10 | 60 |
|  |  |  |  |  |  |  | -4 | 10 | 64 |
|  |  |  |  |  |  |  | -4 | 20 | 46 |
| 3 | Aziz-Zadeh et al. | 2010 | 20 | fMRI | Emotional Voices | Passive | -4 | 4 | 70 |
| 4 | Bengtsson et al. | 2009 | 17 | fMRI | Music | Passive | -4 | 8 | 60 |
|  |  |  |  |  |  |  | -4 | -16 | 60 |
|  |  |  |  |  |  |  | 6 | 11 | 60 |
| 5 | Bestelmeyer et al. | 2014 | 19 | fMRI | Emotional Voices | Active | -3 | 14 | 49 |
| 6 | Binder et al. | 2008 | 26 | fMRI | Speech | Active | -1 | 16 | 49 |
| 7 | Brown and Martinez | 2007 | 11 | fMRI | Music | Active | 2 | -6 | 57 |
| 8 | Callan et al. | 2006 | 16 | fMRI | Music | Passive | -3 | -10 | 75 |
| 9 | Chapin et al. | 2010 | 13 | fMRI | Music | Passive | -3 | -13 | 63 |
|  |  |  |  |  |  |  | -3 | 20 | 53 |
| 10 | Chen et al. | 2008 | 12 | fMRI | Music | Passive | -8 | -4 | 64 |
|  |  |  |  |  |  |  | -2 | 0 | 62 |
|  |  |  |  |  |  |  | 0 | -6 | 69 |
| 11 | Chen et al. | 2012 | 16 | fMRI | Music | Passive | -2 | 8 | 52 |
| 12 | Davis and Johnsrude | 2003 | 12 | fMRI | Speech | Active | -2 | 16 | 46 |
| 13 | Desai et al. | 2010 | 33 | fMRI | Speech | Active | -7 | 2 | 70 |
|  |  |  |  |  |  |  | -9 | -6 | 68 |
|  |  |  |  |  |  |  | -14 | 1 | 68 |
| 14 | Drolet et al. | 2014 | 24 | fMRI | Emotional Voices | Active | -6 | 3 | 53 |
| 15 | Du et al. | 2015 | 16 | fMRI | Speech | Active | -3 | 8 | 55 |
| 16 | Evans et al. | 2016 | 20 | fMRI | Speech | Passive | 2 | 12 | 56 |
| 17 | Gauvin et al. | 2015 | 21 | fMRI | Speech | Active | -6 | 17 | 58 |
| 18 | Gazzola et al. | 2006 | 16 | fMRI | Other | Passive | 0 | -2 | 64 |
| 19 | Geiser et al. | 2008 | 24 | fMRI | Speech | Active | 0 | 21 | 42 |
| 20 | Grahn and Brett | 2007 | 27 | fMRI | Music | Active | -9 | 6 | 60 |
| 21 | Halpern and Zatorre | 1999 | 8 | PET | Imagery | Active | -2 | 3 | 69 |
|  |  |  |  |  |  |  | -2 | -4 | 73 |
| 22 | Halpern et al. | 2004 | 10 | fMRI | Imagery | Active | -6 | -2 | 60 |
| 23 | Herholz et al. | 2012 | 10 | fMRI | Imagery | Passive | -2 | 10 | 58 |
|  |  |  |  |  | Music |  | -8 | 4 | 58 |
| 24 | Herrmann et al. | 2014 | 19 | fMRI | Other | Active | 3 | 17 | 46 |
| 25 | Jäncke et al. | 2003 | 9 | fMRI | Other | Active | -8 | 12 | 44 |
| 26 | Jardri et al. | 2007 | 12 | fMRI | Speech | Passive | 5 | -4 | 53 |
|  |  |  |  |  |  |  | 3 | -7 | 61 |
| 27 | Leaver et al. | 2009 | 9 | fMRI | Imagery | Passive | 5 | -1 | 68 |
|  |  |  | 11 | fMRI | Imagery | Active | -4 | -12 | 69 |
|  |  |  |  |  | Music | Active | 3 | 2 | 57 |
|  |  |  |  |  | Music | Active | -10 | 5 | 51 |
| 28 | Lee et al. | 2012 | 13 | fMRI | Speech | Passive | -3 | 18 | 50 |
| 29 | Liebenthal et al. | 2013 | 25 | fMRI | Speech | Active | -6 | -4 | 57 |
| 30 | Lima, Lavan et al. | 2015 | 74 | VBM | Imagery | Passive | -6 | -13 | 67 |
| 31 | LoCasto et al. | 2004 | 20 | fMRI | Speech | Active | -4 | 17 | 59 |
| 32 | Love et al. | 2006 | 10 | fMRI | Speech | Active | 1 | -14 | 49 |
| 33 | McGettigan et al. | 2015 | 21 | fMRI | Emotional Voices | Passive | -3 | 18 | 51 |
| 34 | McGettigan et al. | 2010 | 17 | fMRI | Speech | Active | -12 | 15 | 45 |
| 35 | Meltzer et al. | 2010 | 24 | fMRI | Speech | Active | -12 | -5 | 57 |
|  |  |  |  |  |  |  | -7 | 0 | 54 |
| 36 | Nastase et al. | 2014 | 17 | fMRI | Other | Passive | -6 | -2 | 60 |
| 37 | Osnes et al. | 2011 | 15 | fMRI | Speech | Passive | 0 | 3 | 66 |
|  |  |  |  |  | Music |  | 0 | 6 | 63 |
| 38 | Osnes et al. | 2012 | 19 | fMRI | Music | Passive | 8 | -22 | 64 |
|  |  |  |  |  |  |  | -2 | 10 | 54 |
|  |  |  |  |  |  |  | -4 | 14 | 52 |
| 39 | Pastor et al. | 2006 | 14 | fMRI | Other | Active | 12 | 12 | 64 |
| 40 | Peelle et al. | 2010 | 40 | fMRI | Speech | Active | -4 | 6 | 64 |
| 41 | Peretz et al. | 2009 | 9 | fMRI | Music | Passive | -4 | -4 | 66 |
| 42 | Rosen et al. | 2011 | 13 | PET | Speech | Passive | 0 | 10 | 54 |
| 43 | Sammler et al. | 2015 | 23 | fMRI | Speech | Active | 9 | 17 | 49 |
| 44 | Saur et al. | 2008 | 33 | fMRI | Speech | Active | -3 | 18 | 54 |
| 45 | Scott et al. | 2004 | 7 | PET | Speech | Passive | -14 | 2 | 70 |
| 46 | Shahin et al. | 2009 | 15 | fMRI | Speech | Active | -6 | 18 | 58 |
| 47 | Shergill et al. | 2001 | 8 | fMRI | Imagery | Passive | 1 | -4 | 59 |
|  |  |  |  |  |  |  | 3 | -7 | 59 |
|  |  |  |  |  |  |  | 3 | -6 | 53 |
|  |  |  |  |  |  |  | 3 | -7 | 59 |
| 48 | Silva-Pereira et al. | 2011 | 27 | fMRI | Music | Passive | 4 | -6 | 56 |
|  |  |  |  |  |  |  | 6 | -6 | 60 |
| 49 | Specht et al. | 2005 | 5 | fMRI | Speech | Active | 4 | 0 | 56 |
| 50 | Tian et al. | 2016 | 18 | fMRI | Imagery | Passive | -8 | 6 | 62 |
|  |  |  |  |  |  |  | 6 | 12 | 52 |
|  |  |  |  |  |  |  | -6 | 18 | 50 |
| 51 | Tremblay and Small | 2011 | 21 | fMRI | Speech | Passive | -7 | 1 | 74 |
| 52 | Trost et al. | 2014 | 18 | fMRI | Music | Active | -8 | -20 | 62 |
| 53 | Venezia et al. | 2012 | 18 | fMRI | Speech | Active | -1 | 2 | 56 |
| 54 | Warren et al. | 2006 | 20 | fMRI | Emotional Voices | Passive | -4 | 0 | 64 |
| 55 | Wild et al. | 2012 | 19 | fMRI | Speech | Active | -6 | 9 | 57 |
| 56 | Wong et al. | 2008 | 11 | fMRI | Speech | Active | -4 | 10 | 57 |
| 57 | Zatorre et al. | 1996 | 12 | PET | Music | Active | 7 | 1 | 67 |
|  |  |  |  |  | Imagery | Active | 4 | 1 | 64 |

Note. In the column Category, ‘Other’ refers to studies that include auditory stimuli such as environmental action sounds (e.g., sound of drinking with a straw), pure tones, clicks, or frequency modulated sounds

**References**

1. Adank, P. and Devlin, J. (2010) On-line plasticity in spoken sentence comprehension: Adapting to time-compressed speech. NeuroImage 49, 1124-1132
2. Adank, P. et al.(2013) Accent imitation positively affects language attitudes. Front. Psychol. 4, 280
3. Aziz-Zadeh, L. et al. (2010) Common premotor regions for the perception and production of prosody and correlations with empathy and prosodic ability. PLoS One 5, e8759
4. Bengtsson, S. L. et al. (2009) Listening to rhythms activate motor and premotor cortices. Cortex 45, 62-71
5. Bestelmeyer, P. E. G. et al. (2014) Adaptation to vocal expressions reveals multistep perception of auditory emotion. J. Neurosci. 34, 8098-8105
6. Binder, J.R. et al. (2008) A comparison of five fMRI protocols for mapping speech comprehension systems. Epilepsia 49, 1980-1997
7. Brown, S. and Martinez, M. J. (2007) Activation of premotor vocal areas during musical discrimination. Brain Cogn. 63, 59-69
8. Callan, D. E. et al. (2006) Song and speech: Brain regions involved with perception and covert production. NeuroImage 31, 1327-1342
9. Chapin, H. L. et al. (2010) Neural responses to complex auditory rhythms: The role of attending. Front. Psychol. 1, 224
10. Chen, J. L. et al. (2008) Listening to musical rhythms recruits motor regions of the brain. Cereb. Cortex 18, 2844-2854
11. Chen, J. L. et al. (2012) Learning to play a melody: An fMRI study examining the formation of auditory-motor associations. NeuroImage 59, 1200-1208
12. Davis, M. H. and Johnsrude, I. S. (2003) Hierarchical processing in spoken language comprehension. J. Neurosci. 23, 3423-3431
13. Desai, R. H. et al. (2010) Activation of sensory-motor areas in sentence comprehension. Cereb. Cortex 20, 468-478
14. Drolet, M. et al. (2014) Recognizing the authenticity of emotional expressions: F0 contour matters when you need to know. Front Hum. Neurosci. 8, 144
15. Du, Y. et al. (2015) Noise differentially impacts phoneme representations in the auditory and speech motor systems. Proc. Natl. Acad. Sci. USA 111, 7126-7131
16. Evans, S. et al. (2016) Getting the cocktail party started: Masking effects in speech perception. J. Cogn. Neurosci. 28, 483-500
17. Gauvin, H. S. et al. (2016) Conflict monitoring in speech processing: An fMRI study of error detection in speech production and perception. NeuroImage 126, 96-105
18. Gazzola, V. et al. (2006) Empathy and the somatotopic auditory mirror system in humans. Curr. Biol. 16, 1824-1829
19. Geiser, E. et al. (2008) The neural correlate of speech rhythm as evidenced by metrical speech processing. J. Cogn. Neurosci. 20, 541-552
20. Grahn, J. A. and Brett, M. (2007) Rhythm and beat perception in motor areas of the brain. J. Cogn. Neurosci. 19, 893-906
21. Halpern, A. R. and Zatorre, R. J. (1999) When that tune runs through your head: A PET investigation of auditory imagery for familiar melodies. Cereb. Cortex 9, 697-704
22. Halpern, A. R. et al. (2004) Behavioral and neural correlates of perceived and imagined musical timbre. Neuropsychologia 42, 1281-1292
23. Herholz, S. C. et al. (2012) Neuronal correlates of perception, imagery, and memory for familiar tunes. J. Cogn. Neurosci. 24, 1382-1397
24. Herrmann, B. et al. (2014) Supplementary motor area activations predict individual differences in temporal-change sensitivity and its illusory distortions. NeuroImage 101, 370-379
25. Jäncke, L. et al. (2003) Focused attention in a simple dichotic listening task: an fMRI experiment. Cogn. Brain Res. 16, 257-266
26. Jardri, R. et al. (2007) Self awareness and speech processing: An fMRI study. NeuroImage 35, 1645-1653
27. Leaver, A. M. et al. (2009) Brain activation during anticipation of sound sequences. J. Neurosci. 29, 2477-2485
28. Lee, Y. et al. (2012) Categorical speech processing in Broca’s area: An fMRI study using multivariate pattern-based analysis. J. Neurosci. 32, 3942-3948
29. Liebenthal, E. et al. (2013) Neural dynamics of phonological processing in the dorsal auditory stream. J. Neurosci. 33, 15414-15424
30. Lima, C. F. et al. (2015) Feel the noise: Relating individual differences in auditory imagery to the structure and function of sensorimotor systems. Cereb. Cortex 25, 4638-4650
31. LoCasto, P. C. et al. (2004) An fMRI investigation of speech and tone segmentation. J. Cogn. Neurosci. 16, 1612-1624
32. Love, T. et al. (2006) A functional neuroimaging investigation of the roles of structural complexity and task-demand during auditory sentence processing. Cortex 42, 577-590
33. McGettigan, C. et al. (2015) Individual differences in laughter perception reveal roles for mentalizing and sensorimotor systems in the evaluation of emotional authenticity. Cereb. Cortex 25, 246-257
34. McGettigan, C. et al. (2010) Neural correlates of sublexical processing in phonological working memory. J. Cogn. Neurosci. 23, 961-977
35. Meltzer, J. A. et al. (2010) Neural aspects of sentence comprehension: Syntactic complexity, reversibility, and reanalysis. Cereb. Cortex 20, 1853-1864
36. Nastase, S. et al. (2014) Uncertainty in visual and auditory series is coded by modality-general and modality-specific neural systems. Hum. Brain Mapp. 35, 1111-1128
37. Osnes, B. et al. (2011) Effective connectivity analysis demonstrates involvement of premotor cortex during speech perception. NeuroImage 54, 2437-2445
38. Osnes, B. et al. (2012) Stimulus expectancy modulates inferior frontal gyrus and premotor cortex activity in auditory perception. Brain Lang. 121, 65-69
39. Pastor, M. A. et al. (2006) The neural basis of temporal auditory discrimination. NeuroImage 30, 512-520
40. Peelle, J. E. et al. (2010) Neural processing during older adults’ comprehension of spoken sentences: Age differences in resource allocation and connectivity. Cereb. Cortex 20, 773-782
41. Peretz, I. et al. (2009) Musical lexical networks: The cortical organization of music recognition. Ann. N. Y. Acad. Sci. 1169, 256-265
42. Rosen, S. et al. (2011) Hemispheric asymmetries in speech perception: Sense, nonsense and modulations. PLoS One 6, e24672
43. Sammler, D. et al. (2015) Dorsal and ventral pathways for prosody. Curr. Biol. 25, 3079-3085
44. Saur, D. et al. (2008) Ventral and dorsal pathways for language. Proc. Natl. Acad. Sci. USA 105, 18035-18040
45. Scott, S. K. et al. (2004) A positron emission tomography study of the neural basis of informational and energetic masking effects in speech perception. J. Acoust. Soc. Am. 115, 813-821
46. Shahin, A. J. et al. (2009) Neural mechanisms for illusory filling-in of degraded speech. NeuroImage 44, 1133-1143
47. Shergill, S. S. et al. (2001) A functional study of auditory verbal imagery. Psychol. Med. 31, 241-253
48. Silva-Pereira, C. et al. (2011) Music and emotions in the brain: Familiarity matters. PLoS One 6, e27241
49. Specht, K. et al. (2005) “Soundmorphing”: A new approach to studying speech perception in humans. Neurosci. Lett. 384, 60-65
50. Tian, X. et al. (2016) Mental imagery of speech implicates two mechanisms of perceptual reactivation. Cortex, 77, 1-12
51. Trembley, P. and Small, S. L. (2011) From language comprehension to action understanding and back again. Cereb. Cortex 21, 1166-1177
52. Trost, W. et al. (2014) Getting the beat: Entrainment of brain activity by musical rhythm and pleasantness. NeuroImage 103, 55-64
53. Venezia, J. H. et al. (2012) Response bias modulates the speech motor system during syllable discrimination. Front. Psychol. 3, 157
54. Warren, J. W. et al. (2006) Positive emotions preferentially engage an auditory-motor ‘mirror’ system. J. Neurosci. 26, 13067-13075
55. Wild, C. J. et al. (2012) Effortful listening: The processing of degraded speech depends critically on attention. J. Neurosci. 32, 14010-14021
56. Wong, P. C. M. et al. (2008) Cortical mechanisms of speech perception in noise. J. Speech Lang. Hear. Res. 51, 1026-1041
57. Zatorre, R. J. et al. (1996) Hearing in the mind’s ear: A PET investigation of musical imagery and perception. J. Cogn. Neurosci. 8, 29-46
58. Adank, P.
